# Morin promotes prostate cancer cells chemosensitivity to paclitaxel through miR-155/GATA3 axis

**DOI:** 10.18632/oncotarget.18133

**Published:** 2017-05-24

**Authors:** Bin Li, Xunbo Jin, Huilin Meng, Bo Hu, Tao Zhang, Jiang Yu, Shaoan Chen, Xudong Guo, Weiguo Wang, Wei Jiang, Jin Wang

**Affiliations:** ^1^ Minimally Invasive Urology Center, Shandong Provincial Hospital Affiliated to Shandong University, Jinan 250021, Shandong, China; ^2^ Department of Urology, Jining No.1 People's Hospital, Jining 272011, Shandong, China; ^3^ Department of Urology, Dongying People's Hospital, Dongying 257091, Shandong, China; ^4^ Department of Urology, Shandong Provincial Qianfoshan Hospital Affiliated to Shandong University, Jinan 250014, Shandong, China; ^5^ School of Basic Medical Sciences, Shandong University, Jinan 250012, Shandong, China

**Keywords:** prostate cancer, morin, paclitaxel-resistance, microRNA (miR)-155, GATA binding protein 3 (GATA3)

## Abstract

Paclitaxel is a first-line microtubule-stabilizing drug in treating prostate cancer. However, most patients develop resistance and experience relapse. Morin (3,5,7,20,40-pentahydroxyflavone) is an anti-tumor flavonoid in a numerous types of cancer cells including breast, ovarian and lung cancers. We therefore researched the effects of morin as an adjuvant to paclitaxel in in treating DU145 and PC-3 cells *in vitro* and DU145 derived prostate cancers in nude mice models. The chemosensitivities of these cells to the treatments of morin and paclitaxel were tested through viability assays utilizing cell counting kit 8 (CCK-8) and apoptosis assays through flow cytometry analyses. MicroRNA (miRNA) microarray was employed to determine the changes in miRNA profile of morin treated DU145 cells. The results from microarrays were further certified by quantitative real-time reverse transcription-PCR (qRT-PCR). The underlying targets of miR-155 were verified using luciferase assays followed by Western blot assays. In the results, morin was capable of repressing the cell viabilities in the paclitaxel-treated cells. MiR-155might be an effective target that can be down-regulated in morin-treated cells. We also discovered that GATA binding protein 3 (GATA3) was directly repressed by miR-155, and the treatment of morin reversed the expression of GATA3. In conclusion, morin might be a potential adjuvant of paclitaxel in treating prostate cancer through regulating miR-155/GATA3 axis.

## INTRODUCTION

Paclitaxel, an alkaloid originally discovered in pacific yew tree, has long been used as a first-line treatment of prostate cancer. The antitumor effect of paclitaxel has been attributed to mitotic arrest, in which microtubule is stabilized to prevent chromosome segregation and cytokinesis [1]. However, most patients rapidly develop resistance to paclitaxel and experience relapse [2]. Therefore, it is important to develop new methods to conquer the drug resistance. By now, it is clear that alterations in the expressions of microtubule subtypes and the subsequent cell signaling could influence the affinity of the drug and the downstream response of the cell [3]. Hence, combination therapy might be efficient to prevent drug resistance through altering the microtubule expressions.

MicroRNAs (miRNAs) are small non-coding RNAs (19-25 nt) that modulate the translation of mRNAs through binding to its 3’-untranslated region (UTR) [4]. Studies have shown that each tumor type has a distinct miRNA profile that correlates to their relevant subtypes, patient survivals and treatment responses in different cancers [5]. This provides a convenient method to understand the efficacies of drugs on tumor cells and their molecular mechanisms. MiR-155 is a multi-functional miRNA that participates in innate and acquired immune responses and hematopoiesis [6, 7]. MiR-155 dysregulation has been reported in numerous malignant tumors including leukemia, breast and lung cancers [8, 9]. Recent data has shown that miR-155 can be markedly overexpressed in prostate cancer cells [10, 11]. Researches have demonstrated that miR-155 could promote cell proliferation, enhance tumor growth and inhibit apoptosis through suppressing the expression of proteins like TP53INP1, VHL, PIK3RI [12–14]. Nevertheless, the understandings of the mechanisms of miR-155 in promoting the progression of cancers, and researches investigating the effects of miR-155 to prostate cancer are limited.

Morin (3,5,7,20,40-pentahydroxyflavone) is a flavonoid isolated from figs and other members of *Moraceae* family. In recent studies, morin has exhibited effects on regulating cell survival and proliferation-related genes, promoting apoptosis and chemo-sensitivity in multiple cancer cell lines [15–17]. Researches have shown that morin can upregulate intrinsic apoptosis pathways [15, 17, 18]. In breast cancer cells, 50 μM of morin was effective in reverting their malignant phenotypes [19]. Therefore, we hypothesized that 50 μM of morin can be an effective adjuvant of paclitaxel in delaying the development of drug resistance. Experiments *in vivo* and *in vitro* showed that morin increased the chemo-sensitivity to paclitaxel. The results of miRNA microarray revealed that miR-155 was significantly repressed by treatment of morin. GATA binding protein 3(GATA3) was significantly downregulated by miR-155.

## RESULTS

### Morin promotes chemo-sensitivity of prostate cancer cells to paclitaxel

**Figure 1:**
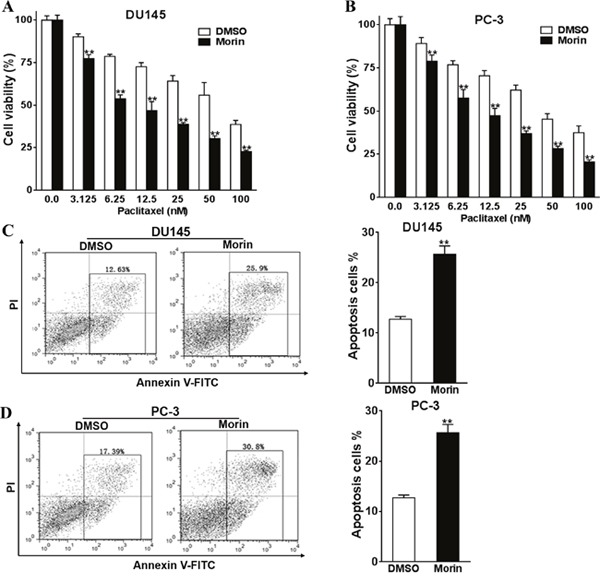
Morin promotes prostate cancer cells chemosensitivity to paclitaxel **(A, B)** The cell viabilities of DU145 and PC-3 cells to morin (50 μM) and paclitaxel (0-100 nM) were evaluated using CCK-8 assay after 48 h of incubation. **(C, D)** The apoptosis levels of DU145 and PC-3 cells to the treatment of morin (50 μM) and paclitaxel (50 nM) were determined by flow cytometry. All of the experiments were triplicated and data were shown in mean±SD, and the data were shown as the percentage compared to the DMSO control. Student T tests were used to verify the significance of differences between two groups. ** p<0.01, indicate significant difference compared to DMSO control.

DU145 and PC-3 cell lines are standard models that have been widely used in studying prostate cancers. Hence they are more universal comparing to cell lines derived from patients. To evaluate the cytotoxicity of morin as an adjuvant to paclitaxel, we firstly evaluated the viabilities of DU145 and PC-3 cells in the environment of morin and paclitaxel using CCK-8 kit. In the experiments, cells were cultured in the medium containing 50 μM morin dissolved in DMSO and a gradient of paclitaxel (0-100 nM) for 48 h (Figure 1) and 72 h (Supplementary Figure 1). The same amount of DMSO was treated in control groups. The cytotoxicity of 50 μM morin alone was limited within 48 h of treatment (Figure 1A and 1B; all P < 0.01). The cell viabilities of both cell lines were influenced only after 72 h treatment comparing with the DMSO control (Supplementary Figure 1A and 1B; all P < 0.01). However, when paclitaxel was treated at the same time, the viabilities of morin-treated groups in both of the cell lines were significantly lower than DMSO control groups in both the time points of 48 h and 72 h (Figure 1A and 1B; Supplementary Figure 1A and 1B; all P < 0.01). These results were then verified by measuring relative apoptosis rates using flow cytometry in PC-3 and DU145 cell lines, in which cells were treated with 50 μM morin and 50 nM paclitaxel for 48 h. Similar to the results in viability assay, morin led to significant increase in cell apoptosis compared with DMSO treatment in both of the cell lines (Figure 1C and 1D; both P < 0.01).

### Morin promotes chemo-sensitivity of prostate cancer cells to paclitaxel *in vivo*

**Figure 2:**
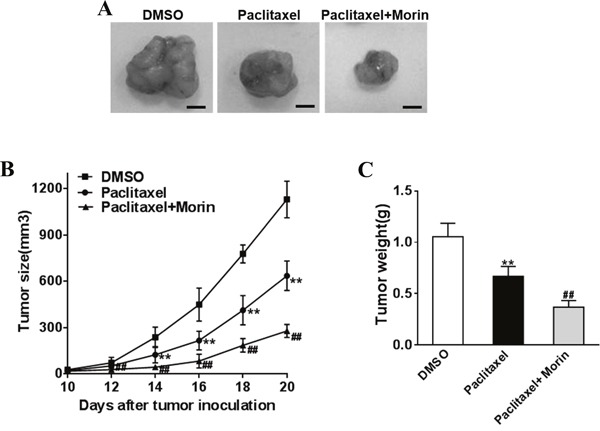
Morin enhances the sensibility of prostate cancer cells to paclitaxel *in vivo* The DU145 cells (5 × 10^6^) were suspended in serum-free 1640 medium (100 μL) and were subcutaneously injected into each side of posterior flank of the nude mice (n=4 for each group at indicated time point), then paclitaxel (50 mg/kg) and morin (50 mg/kg) was added or not by tail vein injection. **(A-C)** Tumors were measured every two days since they were apparently seen and the volumes were calculated using the following formula: volume = 0.5 × Length × Width^2^. Tumors were excised and weighed after 20 days with representative pictures of tumors shown (Bar=10mm). All of the experiments were triplicated and data were shown in mean±SD. Student T tests were used to verify the significance of differences between two groups. One or two-way ANOVA analyses was employed for analyzing statistical differences between multiple groups. ** p<0.01, indicate significant difference compared to DMSO group. ## p<0.01, indicate significant difference compared to Paclitaxel group.

We investigated the inhibitory function of morin as an adjuvant to paclitaxel in treating DU145 derived prostate cancer in nude mice models, in which nude mice were divided into paclitaxel treated, morin and paclitaxel treated and DMSO control groups (4 nude mice for each group at indicated time point). DU145 cells were injected into both sides of posterior flank of nude mice. Paclitaxel (50 μg/kg) and morin (50 mg/kg) were injected by tail vein injection every day for 15 days. Since the day of injection, the tumor sizes were determined every two days, and tumors were excised and weighed after 20 days (Figure 2A–2C). Both the data of tumor size and weight indicated that morin significantly promoted the anti-tumor function of paclitaxel during the process of prostate cancer development *in vivo*.

### Morin inhibits miR-155 expression in prostate cancer cells

**Figure 3:**
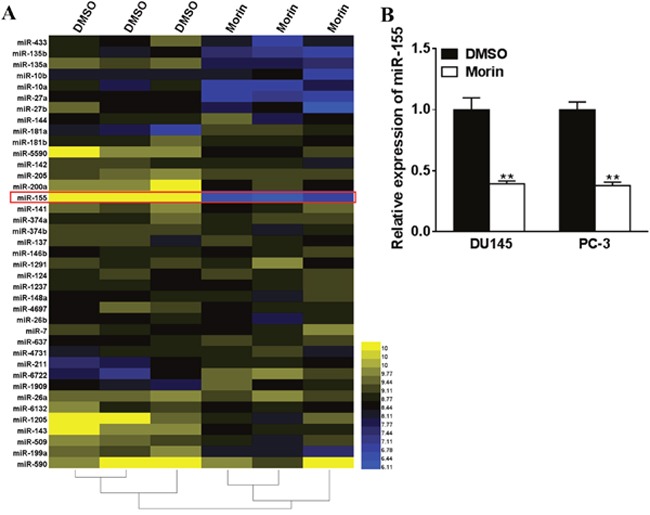
Morin inhibites miR-155 expression in prostate cancer cells **(A)** Morin (50 μM) or DMSO were added into the medium of DU145 cells, and after 24 h of incubation, the levels of miRNAs were checked by microarray. **(B)** DU145 cells were incubated with 50 μM morin or DMSO, and after 24 h, the intracellular concentrations of miRNAs were checked by qRT-PCR. All of the experiments were triplicated and data were shown in mean±SD. Student T tests were used to verify the significance of differences between two groups. ** p<0.01, indicate significant difference compared to DMSO group.

The miRNA profiles of morin and DMSO treated DU145 cells was analyzed to understand how prostate cancer cells were inhibited by morin. A total of 39 microRNAs were found to be altered in morin treated cells, with the most significant one being miR-155 (Figure 3A). Then we assessed the effect of morin on the intracellular concentrations of miR-155 in DU145 and PC-3 cell by qRT-PCR. The results revealed that the transcription of miR-155 was both reduced in morin-treated cells compared with control cells (Figure 3B, both P < 0.01).

### MiR-155 silencing promoted the chemosensitivities to paclitaxel

**Figure 4:**
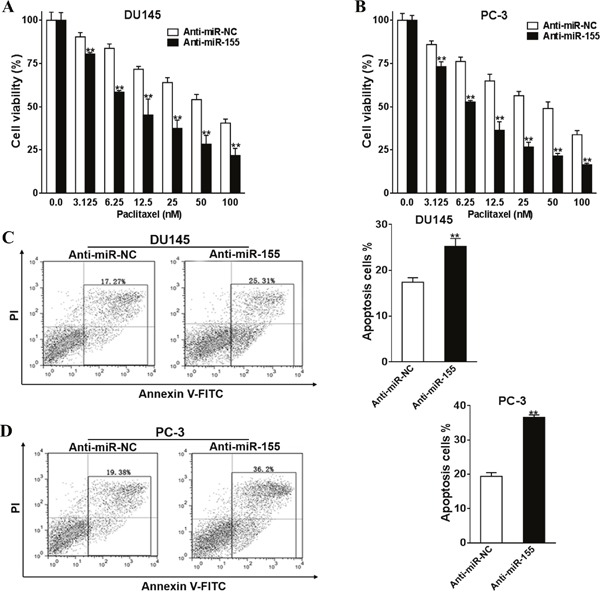
Silence of miR-155 promotes prostate cancer cells chemosensitivity to paclitaxel Anti-miR-155 inhibitor (Anti-miR-155) or control anti-sense RNA (Anti-miR-NC) was transfected into DU145 and PC-3 cells, and were treated with paclitaxel (0-100 nM). After 48 h, CCK-8 assay was used to detected cell viability. **(A, B)** Anti-miR-155, or Anti-miR-NC were transfected into DU145 and PC-3 cells and were treated with 50 nM paclitaxel. Cell apoptosis was analyzed by flow cytometry as described **(C, D)**. All of the experiments were triplicated and data were shown in mean±SD, and the data were shown as the percentage compared to the DMSO+Anti-miR-NC control. Student T tests were used to verify the significance of differences between two groups. ** p<0.01, indicate significant difference compared to the DMSO +Anti-miR-NC control.

The function miR-155 was silenced in DU145 and PC-3 cells to understand its functions in paclitaxel treatment. Firstly, we silenced miR-155 by transfecting anti-miR-155 into DU145 and PC-3 cells. Anti-miR-NC was transfected in the control group. The transfected cells were incubated with paclitaxel (0-100 nM) for 24 h, and CCK-8 kit was subsequently employed to determine the viabilities of these cells. In both cell lines, the viability of miR-155 silenced cells was lower than the control groups (Figure 4A and 4B, all P < 0.01). Apoptosis assays showed that miR-155 silenced cells exhibited elevated percentages of apoptosis cells compared with control cells (Figure 4C and 4D, both P < 0.01).

### Overexpression of miR-155 reverses the effects of morin on tumor cells

**Figure 5:**
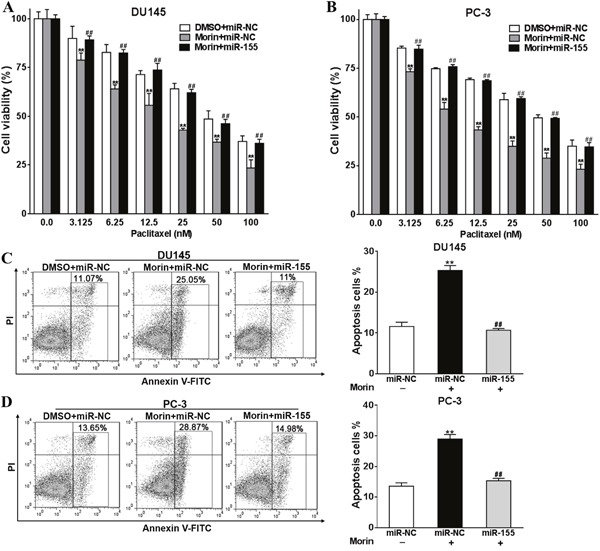
Overexpression of miR-155 reverses the roles of morin in promoting prostate cancer cells sensitivity to paclitaxel **(A, B)** DU145 or PC-3 cells were transfected with miR-155 mimics, or control miR-NC, and were treated with 50 μM morin or DMSO and 0, 3.125, 6.25, 12.5, 25, 50, 100 nM paclitaxel. After 48h, CCK-8 assay was used to detected cell viability. **(C, D)** DU145 or PC-3 cells were transfected with miR-155 mimics, or control miR-NC, and were treated with 50 μM morin or DMSO and 50 nM paclitaxel. After 48h, cell apoptosis was analyzed by flow cytometry as described. All of the experiments were triplicated and data were shown in mean±SD, and the data were shown as the percentage compared to the DMSO+miR-NC control. Student T tests were used to verify the significance of differences between two groups. One or two-way ANOVA analyses was employed for analyzing statistical differences between multiple groups. ** p<0.01, indicate significant difference compared to DMSO+miR-NC control. ## p<0.01, indicate significant difference compared to Morin +miR-NC group.

To understand if miR-155 is directly regulated by morin when it acts as an adjuvant to paclitaxel, miR-155 was overexpressed in DU145 and PC-3 cells. To do this, miR-155 mimics were transfected to increase the intracellular level of miR-155, and miR-NC were transfected as control. Transfected cells were then incubated with or without 50 μM morin and 0-100 nM paclitaxel at 37°C. After 48 h of treatments, the viabilities of cells treated with morin were notably decreased when comparing with the control cells treated with DMSO in both cell lines (Figure 5A and 5B; all P < 0.01). However, the transfection of miR-155 mimics rallied the degree of apoptosis to the level similar to the control group (Figure 5A and 5B; all P < 0.01). These results were consistent with them from apoptosis assays, which were performed on cells pre-treated with 50 nM paclitaxel. In the experiments, the transfection of miR-155 in morin treated cells reversed the degree of apoptosis to a lower level (Figure 5C and 5D, all P < 0.01).

### GATA3 was a downstream effector of miR-155

**Figure 6:**
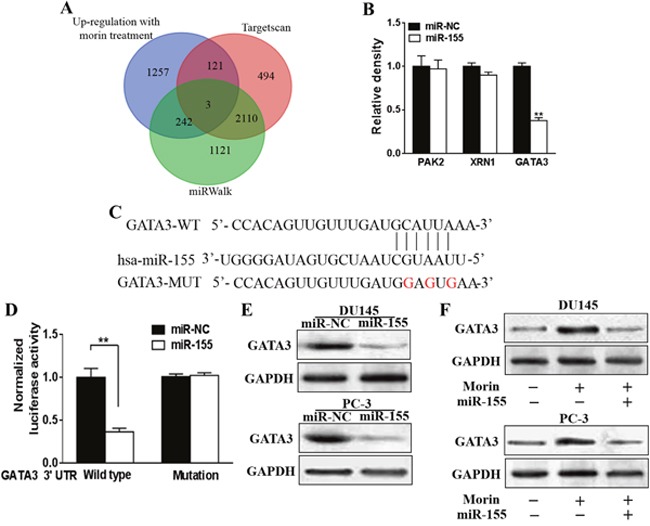
Morin induced miR-155 directly targets GATA3 **(A)** Venn diagram was used to represent the predicted targets of miR-155 in Targetscan and miRWalk which were also up-regulated with morin treatment. **(B)** DU145 cells were transfected with miR-155 mimics, or control miR-NC, and the relative expression levels of predicted targets were analyzed by qRT-PCR. **(C)** Putative seed-matching sites or mutant sites (red) between miR-155 and 3'-UTR of GATA3. **(D)** Luciferase reporter assay was performed on DU145 cells to detect the relative luciferase activities of WT and mut GATA3 reporters. Renilla luciferase vector was used as an internal control. Student T tests were used to verify the significance of differences between two groups. All of the experiments were triplicated and data were shown in mean±SD. ** p<0.01. **(E)** DU145 or PC-3 cells were transfected with miR-155 mimics, or control miR-NC. After 48 h, total proteins of miR-155 and miR-NC-expression cells were subjected to western blotting and detected for GATA3 expression levels. GAPDH expression was served as an internal control. **(F)** DU145 or PC-3 cells were transfected with miR-155 mimics, or control miR-NC, and were treated with 50 μM morin or DMSO. After 48 h, total proteins of the cells were subjected to western blotting and detected for GATA3 expression levels.

We performed *in silico* analyses to determine the underlying targets of miR-155 through Targetscan (http://www.targetscan.org/vert_71/) and miRWalk (http://zmf.umm.uni-heidelberg.de/apps/zmf/mirwalk2/, Figure 6A). Among the 5348 genes evaluated, we found that 3 genes, PAK2, XRN1 and GATA3 contained most potential as targets of miR-155 (Figures 6A and 6B). The transcription of these proteins in miR-155 mimic transfected DU145 cells and control cells were then analyzed through qRT-PCR. The expressions of all of the three proteins were suppressed in different degree. However, the suppression effect of miR-155 mimic to PAK2 and XRN1 were limited comparing to its effects to GATA3. (Figure 6B; P < 0.01). To understand if GATA3 is directly regulated by miR-155, the 3’-UTR of GATA3 was mutated to a sequence that cannot complement miR-155, and Luciferase reporter assays were employed in DU145 cell lines transfected with miR-155 mimics or miR-NC (Figure 6C). We observed that the luciferase activities of wild type GATA3 3’-UTR, but not mutated GATA3 3’-UTR, were notably suppressed in cells with enhanced miR-155 mimics (Figure 6D, P < 0.01). In spite of this, the expression of GATA-3 in these cells were analyzed by Western blotting, in which miR-155 mimics decreased the GATA3 expression level in both cell lines in comparison to the control group (Figure 6E).

### Morin assisted the antitumor activity of paclitaxel through miR-155/GATA3 axis

We then evaluated the role of GATA3 in prostate cancer and if it can be regulated by the treatments of morin and paclitaxel. Firstly, the expressions of GATA3 were significantly decreased in prostate cancer tissues (Supplementary Figure 3, P<0.01), and the overexpression of GATA3 has been demonstrated to increase the chemosensitivity of DU145 and PC-3 cells to paclitaxel (Supplementary Figure 4, P<0.01). We subsequently evaluated the influences of morin and paclitaxel on the expressions of GATA3 in DU145 and PC-3 cell lines. The treatment of paclitaxel or morin alone elevated the expression of GATA3, and morin enhanced the effects of paclitaxel (Supplementary Figure 2, Figure 6F). The next step was to evaluate how morin regulated the expression of miR-155. The cellular level of GATA3 in morin treated miR-155 mimic transfected or control DU145 and PC-3 cells were compared through western blotting, in which the elevated expressions of GATA3 under the treatment of morin were inhibited by miR-155 mimics. We further demonstrated that the relative expression levels of all of the downstream modulators of GATA3 including ZEB2, TGFB1 and MDM2 were inhibited (Supplementary Figure 5, p<0.01). Hence, it can be said that there might be a miR-155/GATA3 axis involved in the treatment of morin.

## DISCUSSION

Prostate cancer is one of the leading causes of cancer death in male worldwide. Though it is curable in its early stage, the median survival in man with advanced metastatic castration-resistant prostate cancer is less than 2 years [20]. Paclitaxel is the only Food and Drug Administration (FDA) approved chemotherapy that is effective in extending the overall survival of prostate cancer patients [21]. This can be ascribed to the fact that tumors easily acquire resistance to paclitaxel and its derivatives (docetaxel and cabazitaxel) even with the assistance of various adjuvants [21–23]. Recent study has shown that prostate cancer even becomes more aggressive and invasive when it acquires paclitaxel resistance [24]. Hence it is crucial to develop new adjuvants to overcome the drug resistance in prostate cancer. In the present study, we demonstrate that morin may increase the chemo-sensitivity of prostate cancer cells to paclitaxel treatment through modulating the expression of miR-155. We also demonstrate that GATA3 may be an important protein involved in the functions of miR-155 in tumor progression.

Morin is a flavonoid isolated from plants of Moraceae family, and contains two aromatic rings linked by an oxygen-containing heterocycle [25]. The anti-tumor effects of morin are widely discovered in a variety of cancers especially in breast cancer and leukemia [15, 18, 26–28]. It has been shown that morin is capable of inhibiting the proliferation and promoting the apoptosis in prostate cancer cell line LNCaP [29, 30], which is consistent with our results that morin repressed the cell viability and enhanced apoptosis of both PC-3 and DU145 cell lines under the treatment of paclitaxel.

Studies have demonstrated that morin induces apoptosis in cancer cell lines through intrinsic apoptosis pathways [17, 18, 26, 27]. The decreased mitochondria potential, the increased expression of caspase-3 and -9, p21 and Bad proteins and the decreased expression of Bcl-xL and STAT3 proteins were widely discovered [17, 18, 26]. Yet, mechanisms underlying the regulation of morin on cell viabilities and apoptosis remain unclear. In this research, we found 39 microRNAs that were dysregulated after the treatment of morin including miR-143, miR-146b, and miR-155. The downregulation of miR-143 and miR-146b has been shown in all types of prostate tumors, which is consistent with our results [31]. However, the inhibitory effects of morin to these two microRNAs were not as effective as miR-155 in our study. The suppression of miR-155 was also discovered the study of the influence of several isoflavones from soy on prostate cancer cell lines, where miR-155, miR-208b, miR-211, miR-376a and miR-411 were found to be downregulated by the isoflavones by more than three to five fold than control cells [32]. The upregulation of miR-155 has been discovered in breast cancer cell lines [10, 11]. In prostate cancer cells, miR-155 was capable of upregulating annexin 7, which is a substrate of a variant of kinases including protein kinase C involved in cell growth, proliferation, apoptosis and migration [10]. Hence, the function of miR-155 in the development of prostate cancer might be underestimated.

The study of the targets of miR-155 in prostate cancer is still limited comparing with other cancers. For instance, in breast cancer at least 147 validated targets have been identified [33]. However, in prostate cancer, only a few downstream effectors of miR-155 have been discovered yet. In present study, we found that GATA3 can be directly regulated by miR-155. GATA3 has been shown highly expressed in normal human and mouse prostates, which is involved in the regulation of prostate-specific antigen (PSA) genes [34, 35]. However, previous researches have shown that its expression is negative in prostate cancer cell lines [34]. In Pten-deficient prostate tumors, the expression of GATA3 is gradually lost during its progression [36]. This is consistent with our results (Supplementary Figure 3). In one research 2-fold of overexpression of GATA3 was effective enough to limit the viabilities of PC-3 cells [37]. In our research we demonstrated that the treatment of morin and/or paclitaxel recovered the expression of GATA3 through inhibiting the expression of miR-155. Researches have shown that ZEB2, TGFB1, MDM2 are important downstream effectors that can be inhibited by GATA3 in breast cancer [38]. In our study, the treatment of morin alone raised the expression of GATA3, and decreased the intracellular levels of ZEB2, TGFB1, MDM2. Therefore it is possible that there is a miR-155/GATA3 axis involved in the antitumor effects of morin as an adjuvant to paclitaxel in treating prostate cancer.

In conclusion, we hereby demonstrate that morin may improve the chemo-sensitivity of prostate cancer cells to paclitaxel through restoring the miR-155-suppressed expression of GATA3. Hence, morin may be a promising adjuvant to paclitaxel in the treatment of prostate cancer.

## MATERIALS AND METHODS

### Cell lines

DU145 and PC-3 prostate cancer cell lines were purchased from Xinyu Biotechnology Co. Ltd (Shanghai, China). All cell lines were cultured in RPMI 1640 (GIBCO-BRL, Grand Island, NY) medium supplemented with 10% fetal bovine serum (FBS), 100 U/ml penicillin, and 100 μg/ml streptomycin in humidified air at 37°C with 5% CO_2_.

### Reagents

Concentrated morin (Sigma-Aldrich, MO, USA)) or paclitaxel (Sigma-Aldrich) were dissolved in DMSO and then diluted into the cultural medium with desired concentration, in which the concentration of DMSO was less than 0.05%.

### miRNA transfection

MiR-155 mimics, anti-miR-155 (Anti-miR-155), miR-NC and anti-sense RNA (Anti-miR-NC) were transfected into DU145 and PC-3 cell lines through Ambion® Pre-miR™ miRNA Starter Kit (Thermo Scientific, Wilmington, DE).

### Cell viability assays

Cells were seeded and cultured in 96-well plates for 24 h. Paclitaxel (Sigma Chemicals, St Louis, Mo, USA) was then added into the culture media in the gradient of 0 μM, 3.125 μM, 6.25 μM, 12.5 μM, 25 μM, 50 μM, 100 μM. The cell viability was determined after 48 h of incubation using cell counting kit 8 (CCK-8) (Dojindo Laboratories, Kumamoto, Japan). In the cell viability assay, 10 μl CCK-8 solution was added into each well of the plate. After 4 h of incubation at 37 °C, the cell viabilities were analyzed at dual wave lengths (450 nm and 630 nm). The experiments were repeated for three times.

### Flow cytometry analysis of apoptosis

Cells were cultured in 50 μM morin and 50 nM paclitaxel for 48 h, and then were harvested and centrifuged. The centrifuged cells were then washed twice utilizing cold PBS buffer and resuspended into the concentration of 1×10^6^ cells/mL. Annexin V Apoptosis Detection kit (eBIOscience, San Diego, CA, USA) was utilized for annexin V double staining. Annexin V solution (5 μL) was mixed with100 μL cell suspensions, The mixture were incubated at dark for 15 min, and the cells were washed with 2 mL 1×binding buffer. The cells were again suspended in 200 μL of 1×binding buffer 5 μL of Propidium Iodide Staining Solution was added into solution before flow cytometry. Beckman Coulter Cytomics FC 500 (San Jose, CA, USA) was employed for flow cytometry analysis.

### Tumor formation assay in nude mice

5 × 10^6^ DU145 cells were centrifuged and dispersed in 100 μl serum-free 1640 medium. The suspensions were injected into both sides of posterior flanks in nude mice. 50 μg/kg of paclitaxel and 50 mg/kg of morin were injected through tail vein every day for 20days. Meanwhile, the tumor sizes were measured every two days since tumors can be apparently seen (Volume = 0.5 × Length × Width^2^). Tumors were excised and weighted in the 20^th^ day.

### miRNA microarray

DU145 cells were culture in the medium containing 50 μM morin or the same amount of DMSO (less than 0.05% in the medium), and after 24 h of incubation, the levels of miRNAs were checked by microarray. Total RNAs inside cells were extracted and purified through PureLinkTM Total RNA isolation Kit (Invitrogen, Carlsbed, CA, USA), and the concentration and purity of the extracted RNAs were examined with NanoDrop ND-1000 spectrophotometer (Thermo Scientific). Affymetrix miRNA 4.0 Array and HG133plus2 microarrays were employed in analyzing the miRNA profiles. The signal intensity of 500 miRNA probes in both of the chips were measured, and the raw data were noemalized and analyzed using GenePix1 4000B Microarray Scanner (Thermo Scientific) and PANAGENE software (Thermo Scientific). All of the procedures were triplicated.

### Quantitative real time-PCR

The total RNA of DU145 and PC-3 cell lines was reverse transcribed into first line cDNA with TaqMan Reverse Transcription Reagents (Life Technologies, Carlsbad, CA, USA), which was then subject to Bio-Rad CFX96 real-time PCR System (Bio-Rad, CA, USA). Primers were designed for different targets, and primers designed for GAPDH and U6 were applied as internal controls.:

miR-155:

 Sense: 5’-AGCCTCCCGCTTCGCTCTCT-3’,

 Antisense: 5’-AGCCTCCCGCTTCGCTCTCT-3’,

PAK2:

 Sense: 5’-AGCCTCCCGCTTCGCTCTCT-3’,

 Antisense: 5’-AGCCTCCCGCTTCGCTCTCT-3’,

XRN1:

 Sense: 5’-AGCCTCCCGCTTCGCTCTCT-3’,

 Antisense: 5’-AGCCTCCCGCTTCGCTCTCT-3’,

GATA3:

 Sense: 5’-AGCCTCCCGCTTCGCTCTCT-3’,

 Antisense: 5’-AGCCTCCCGCTTCGCTCTCT-3’,

### Dual-luciferase reporter assay

GATA3 and GATA3 MUT 3’UTR were firstly amplified using primers:

 GATA3 forward: 5’-ATAGCATCGCAGGTAACGCTACGCGGGACG-3’

 GATA3 Reverse: 5’-ATAGCATCGCAGGTAACGCTACGCGGGACG-3’

 GATA3 MUT forward: 5’-ATAGCATCGCAGGTAACGCTACGCGGGACG-3’

 GATA3 MUT Reverse: 5’-ATAGCATCGCAGGTAACGCTACGCGGGACG-3’

GATA3 and GATA3 mute 3’ UTR were recombined upstream of Firefly luciferase in one vector (Promega, Madison, WI, USA). The luciferase reporter vectors and miR-155 mimics or miR-NC were transfected into DU145 and PC-3 cells. Renilla luciferase vector (Promega) was transfected at the same time as internal control. The luciferase activities were detected through Dual-Glo Luciferase assay system (Promega) after 48 h from transfection.

### Western blot

miR-155 mimics, or miR-NC control were transfected into DU145 and PC-3 cells, which were subsequently incubated with morin (50 μM) and paclitaxel (0-100 nM) for 48 h. Proteins Cell lysates of DU145/miR-155, DU145/miR-NC, PC-3/miR-155 and PC-3/miR-NC cells treated with morin or not were then separated by SDS-PAGE. The separated proteins were then electrophoretically transferred onto NC membranes (Sigma). The membranes were blocked with 5% non-fat milk in TBS buffer (Sigma-Aldrich,) overnight. Anti-human GATA3 primary antibody purchased from Abcam (Cambridge, MA, USA) and horseradish peroxidase-conjugated secondary antibody (Abcam) were incubated with the membrane. The antibodies were dissolved in PBS buffer and diluted in the ration of 1:1000. Enhanced Chemiluminescence (ECL) (Sigma-Aldrich) was employed to detect the immunoreactive bands through Chemigenius Bioimaging system (Syngene, Frederick MD, USA.) GADPH (Abcam) was detected as loading control.

### Statistical analysis

All data was analyzed using SPSS version 13.0 (IBM, Armonk, NY, USA). The results were presented in the formation of mean±standard deviation (SD). One or two-way ANOVA analyses was employed for analyzing statistical differences between multiple groups. Student T tests were used to verify the significance of differences between two groups. P < 0.05 was considered as statistical significance.

## SUPPLEMENTARY MATERIALS FIGURES


