# Supplementary material for: Morin promotes prostate cancer cells chemosensitivity to paclitaxel through miR-155/GATA3 axis
Source: Oncotarget. 2017 May 24;8(29):47849–60. doi: 10.18632/oncotarget.18133 (PMC5564610; doi:10.18632/oncotarget.18133)
Supplement: Supplementary file 1 [file oncotarget-08-47849-s001.pdf]

Morin promotes prostate cancer cells chemosensitivity to paclitaxel through miR-155/GATA3 axis

SUPPLEMENTARY MATERIALS

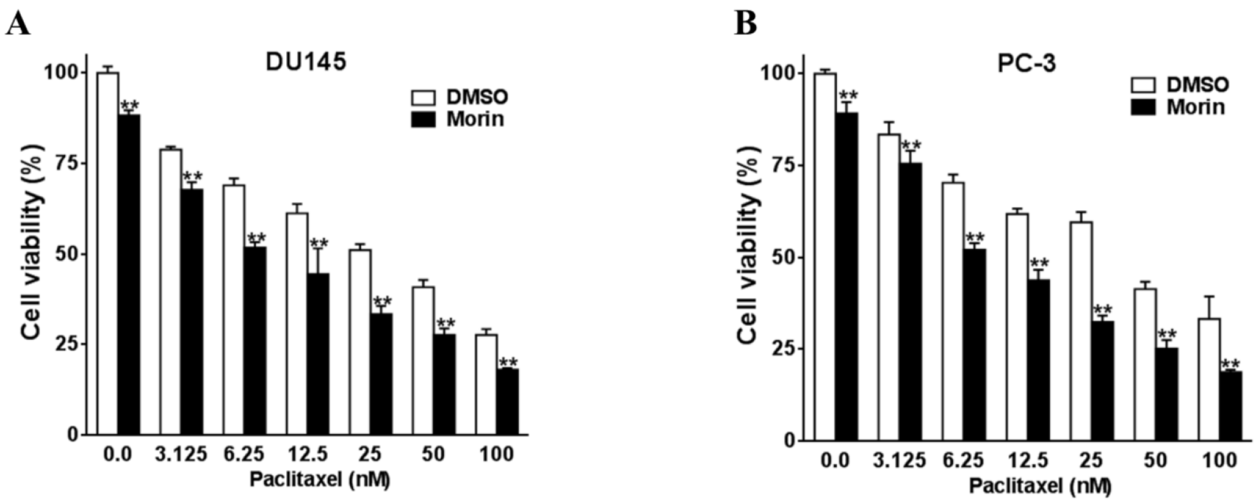

Supplementary Figure 1: Morin promotes prostate cancer cells chemosensitivity to paclitaxel. (A, B) The cell viabilities of DU145 and PC-3 cells to morin (50 μM) and paclitaxel (0-100 nM) were evaluated using CCK-8 assay after 72 h of incubation. Data were presented as mean+SD from three independent experiments with triple replicates per experiment, and the data were shown as the percentage compared to the DMSO control. ** $p<0.01$, indicate significant difference compared to DMSO control.

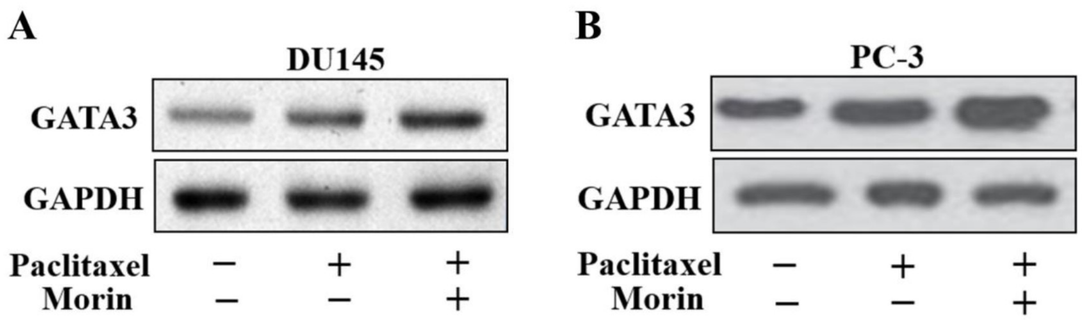

Supplementary Figure 2: Morin induces the upregulation of GATA3 with the treatment of paclitaxel. **(A, B)** The DU145 and PC-3 cells were treated with morin (50 μM) or paclitaxel (50 nM), and after 48 h total proteins of the cells were subjected to western blotting and detected for GATA3 expression levels. GAPDH expression was served as an internal control.

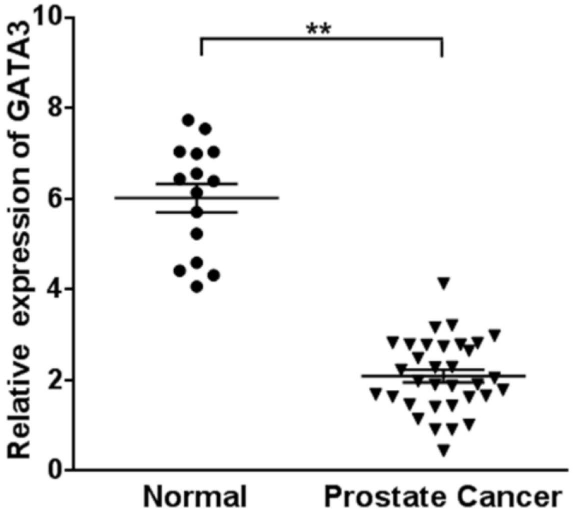

Supplementary Figure 3: GATA3 is downregulated in prostatic cancer. Relative GATA3 expression levels were analyzed by qRT-PCR in normal (n=15) and prostatic cancer tissues (n=33). Data were presented as mean+SD from three independent experiments with triple replicates per experiment, and the data were shown as the percentage compared to the DMSO control. ** $p<0.01$, indicate significant difference compared to normal group.

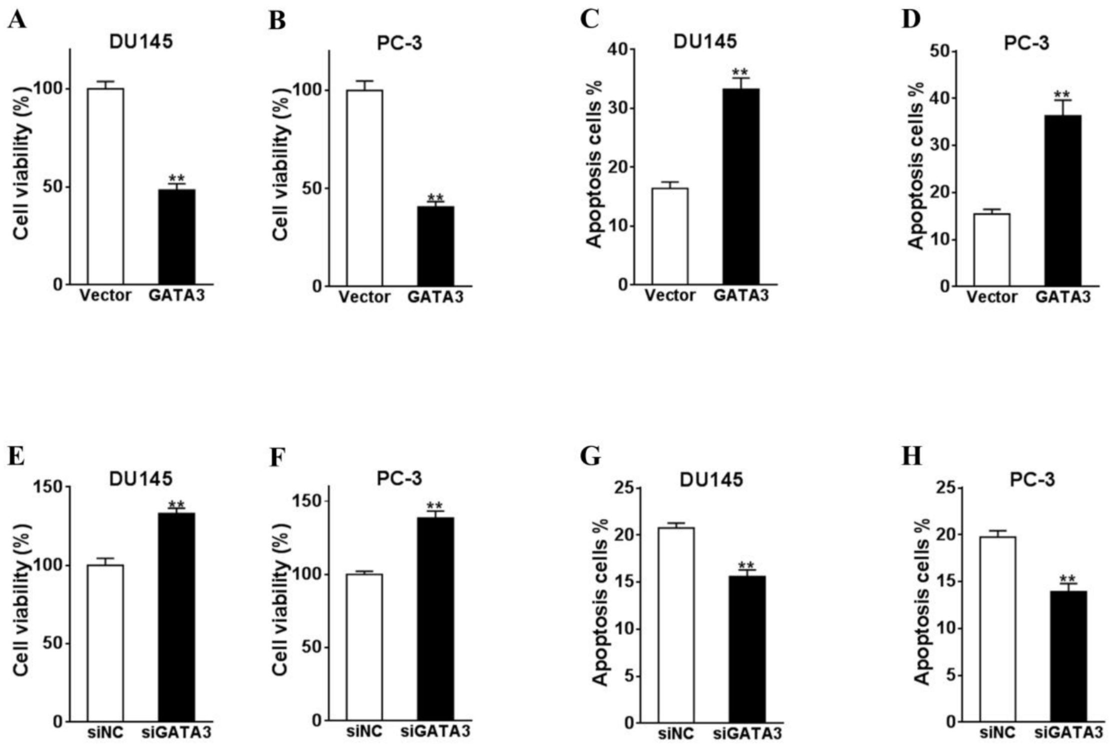

Supplementary Figure 4: GATA3 enhances the chemosensitivity of prostate cancer cells to paclitaxel. **(A, B)** DU145 or PC-3 cells were transfected with GATA3 or control vector, and were treated with 50 nM paclitaxel. After 48h, CCK-8 assay was used to detected cell viability. Data were presented as mean+SD from three independent experiments with triple replicates per experiment, and the data were shown as the percentage compared to the vector control. ** $p<0.01$, indicate significant difference compared to vector control. **(C, D)** DU145 or PC-3 cells were transfected with GATA3 or control vector, and were treated with 50 nM paclitaxel. After 48h, cell apoptosis was analyzed by flow cytometry as described. ** $p<0.01$, indicate significant difference compared to vector control. **(E, F)** DU145 or PC-3 cells were transfected with shGATA3 or mock control, and were treated with 50 nM paclitaxel. After 48h, CCK-8 assay was used to detected cell viability. ** $p<0.01$, indicate significant difference compared to mock control. **(G, H)** DU145 or PC-3 cells were transfected with shGATA3 or mock control, and were treated with 50 nM paclitaxel. After 48h, cell apoptosis was analyzed by flow cytometry as described. ** $p<0.01$, indicate significant difference compared to mock control.

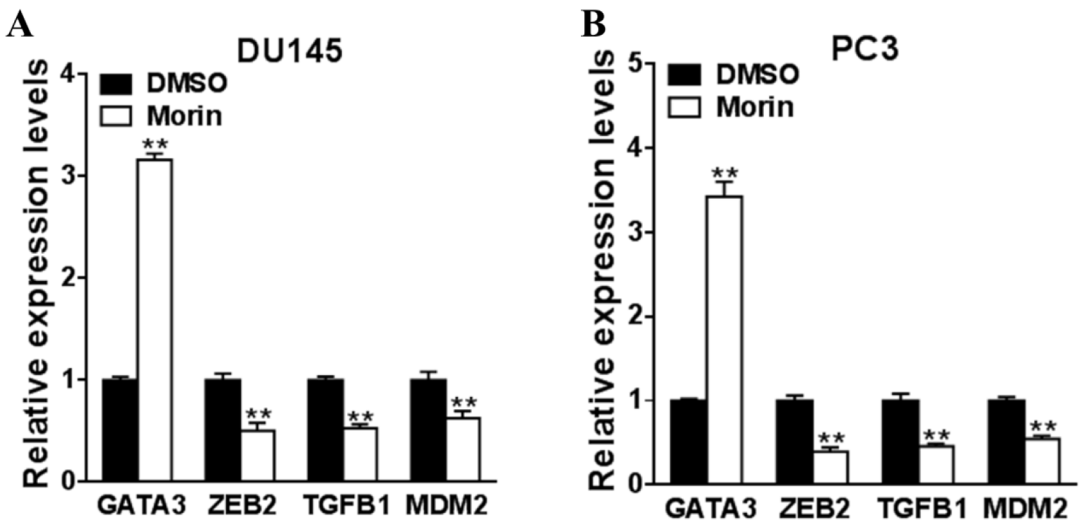

Supplementary Figure 5: Morin influences the downstream genes of GATA3. **(A, B)** The DU145 and PC-3 cells were treated with morin (50 μM) or DMSO, and after 24 h the expression levels of GATA3 and downstream genes (ZEB2, TGFB1, MDM2) were analyzed by qRT-PCR. Data were presented as mean+SD from three independent experiments with triple replicates per experiment, and the data were shown as the percentage compared to the DMSO control. ** p<0.01, indicate significant difference compared to DMSO control.
